# Skin Lightening and Evenness Effects of Oral Supplementation With S‐Acetyl Glutathione in Comparison With Reduced Glutathione: A Double‐Blind, Placebo‐Controlled Clinical Trial

**DOI:** 10.1111/jocd.71223

**Published:** 2026-10-14

**Authors:** Arie Kusumawardani, Lilik Norawati, Silvia Veronica, Alamanda Murasmita, Ervina Rosmarwati

**Affiliations:** ^1^ Department of Dermatology, Venereology and Aesthetics, Faculty of Medicine Universitas Sebelas Maret/Dr. Moewardi General Hospital Surakarta Central Java Indonesia; ^2^ Department of Dermatology, Venereology and Aesthetics Gatot Soebroto Army Central Hospital Jakarta Indonesia

**Keywords:** glutathione, hyperpigmentation, oral supplementation, S‐acetyl glutathione, skin‐lightening

## Abstract

S‐Acetyl Glutathione (SAG) is an advanced glutathione derivative designed to improve stability, bioavailability, and efficacy in skin‐lightening applications. Unlike conventional glutathione formulations, SAG offers great cellular uptake and antioxidative properties, making it a promising candidate for managing hyperpigmentation disorders.

**Purpose:**

This randomized, double‐blind, placebo‐controlled multicenter study evaluates the efficacy and safety of SAG compared to reduced glutathione and placebo in reducing hyperpigmentation, enhancing skin health, and improving patient's satisfaction and physician's assessment.

**Patients and Methods:**

A total of 90 participants with Fitzpatrick skin Type IV and V were randomly assigned to receive oral crystalline form of SAG (200 mg/day), reduced glutathione (500 mg/day), or placebo. The primary outcome was the change in melanin index from Week 0 to Week 12, measured via Mexameter readings. Secondary outcomes included the change of transepidermal water loss (TEWL), pigmentation changes measured via facial skin analyzer, patient's and physician's satisfaction assessments. Exploratory outcomes included change in total glutathione (GSH).

**Results:**

SAG demonstrated better melanin index reduction compared to reduced glutathione and placebo. SAG showed faster reduction in melanin index compared to reduced glutathione and placebo statistically significantly. TEWL showed significant differences at Week 8 and Week 12 between groups. A significant results confirmed in patient's and physician's satisfaction assessment. However no significant pigmentation reduction in all groups, and no statistical significant different in GSH level between groups.

**Conclusion:**

SAG show faster acceleration in reducing melanin index, thus this helps patients with hyperpigmentation received a better result in shorter period, both SAG and reduced glutathione also enhancing skin barrier function, and improving overall skin aesthetics, though SAG has clinically proven, no significancy in pigmentation reduction should be noted. Further studies are recommended to assess its long‐term safety and efficacy in diverse populations.

## 1 Introduction

Pigmentation disorders such as melasma are dermatological conditions characterized by brownish or darker patches compared to the surrounding skin tone [1]. These conditions not only pose aesthetic challenges but also carry significant psychosocial and cultural implications [2]. They are predominantly influenced by complex biochemical pathways, with oxidative stress playing a crucial role [1]. Glutathione (GSH), as one of the primary non‐enzymatic antioxidants in the human body, has garnered considerable attention due to its ability to modulate melanin synthesis by inhibiting tyrosinase activity and directing eumelanin synthesis toward pheomelanin production [3]. However, the therapeutic application of glutathione through oral supplementation faces significant challenges due to its limited bioavailability and susceptibility to enzymatic degradation [4].

Skin lightening has gained considerable attention in both cosmetic and medical fields, driven by cultural preferences for lighter skin tones in various populations [2]. A lighter skin tone has been considered a desirable trait, especially among women of Asian or African descent with Fitzpatrick skin types IV–VI [5, 6]. The higher prevalence of pigmentary disorders in these skin types exacerbates the challenges faced by patients [7]. This cultural phenomenon often motivates individuals to seek skin‐lightening solutions, even when such decisions may conflict with their personal preferences [6, 8].

Realizing this growing demand for bright skin, pharmaceutical companies have responded by developing a range of molecules designed for nutricosmetic purposes. A significant body of research exists on topical depigmenting agents such as glycolic acid, arbutin, kojic acid, vitamin C, vitamin E, and niacinamide, all of which are readily available over the counter [9, 10]. The introduction of newer molecules, including pycnogenol, orchid and marine algae extracts, cinnamic acid, soy, aloesin, and Boswellia, has expanded topical treatment options [11]. However, these agents are limited by their localized effects and potential for adverse reactions at the site of application, prompting a logical progression toward systemic approaches for skin lightening. Systemic agents such as glutathione, tranexamic acid, l‐cysteine peptide, vitamin C, and various plant extracts have gained traction in addressing these limitations [12, 13].

Among systemic agents, glutathione has emerged as a prominent candidate. Recent studies have explored various formulations of glutathione, including topical and oral applications, with varying degrees of effectiveness [14, 15]. Specifically, SAG has been introduced as a more stable form with higher bioavailability that enhances the effectiveness of traditional glutathione treatments [16, 17]. Unlike reduced glutathione, SAG's acetylation enhances its stability, protects it from enzymatic hydrolysis, and facilitates its cellular uptake [16, 17]. Once inside the cytoplasm, SAG is converted to active GSH, exerting potent antioxidant effects. This enhanced bioavailability positions SAG as a great candidate for addressing oxidative imbalance in pigmentation disorders [18].

Despite the growing popularity of glutathione‐based products, a dichotomy persists between anecdotal claims and scientific evidence regarding their safety and efficacy. The Food and Drug Administration (FDA) has expressed concerns over unverified claims associated with intravenous glutathione injections, leading to warnings against their use for off‐label indications such as skin lightening [19]. These concerns underscore the importance of rigorous scientific evaluation to establish the safety and efficacy of glutathione formulations. Therefore, this multicenter study seeks to provide robust clinical data on the safety profile and effectiveness of oral SAG in reducing hyperpigmentation and improving skin tone.

This study aims to evaluate the effectiveness of 12‐week supplementation with 200 mg/day SAG in reducing skin pigmentation compared to reduced 500 mg glutathione and placebo, using melanin index measurements assessed via Mexameter. The research further examines TEWL to evaluate improvements in skin barrier function, pigmentation from facial skin analysis, as well as satisfaction assessment from both patients and physicians, along with systemic GSH level changes to explore the biological effects of supplementation. By integrating these multidimensional outcomes, this study seeks to comprehensively assess the potential of oral SAG in enhancing skin aesthetics and overall skin health.

This study also aims to provide insights into the clinical efficacy of SAG as an advanced glutathione therapy. By addressing gaps in current treatment approaches, this research has the potential to redefine standards for oral intervention with glutathione‐based interventions and inspires further innovations in dermatological science and skincare solutions.

## 2 Methods

### 2.1 Study Design

This randomized, double‐blind, placebo‐controlled multicenter study was conducted in 2024 at the Dermatology and Venereology outpatient clinics of Regional General Hospital Dr. Moewardi, Surakarta. The study enrolled healthy female participants aged 25–50 years with Fitzpatrick skin types IV and V to evaluate the effectiveness and safety of oral SAG as a skin‐lightening agent in comparison to reduced glutathione and placebo. Ethical approval for the study was obtained from the Health Research Ethics Committees of Regional General Hospital Dr. Moewardi, Surakarta (approval number 2.235/XII/HREC/2023). This study was registered with the Indonesia Clinical Research Registry (INA‐CRR) under registration number: INA‐19052025B8A3E39.

### 2.2 Participants

This study employed a total sample approach, selecting participants based on specific inclusion and exclusion criteria to ensure a controlled and homogeneous sample. The inclusion criteria comprised healthy females aged 25–50 years with Fitzpatrick skin types IV and V, who provided informed consent and adhered to specific lifestyle requirements. The sample size was calculated using a standard sample size calculator (https://riskcalc.org/samplesize) to detect a clinically meaningful difference in the primary outcome. Assuming a two‐sided α of 0.05, 80% power, and an expected effect size of *d* = 0.5. Participants were required to spend most of their working hours indoors (minimum 8 h per day) and consistently use sunscreen when outdoors, provided the sunscreen did not contain glutathione or related compounds.

Exclusion criteria were designed to minimize confounding variables. Participants with a personal or family history of melanoma or other skin cancers were excluded, as were those who had used topical skin‐lightening agents or supplements containing glutathione or antioxidants within 1 month prior to enrolment. Pregnant or lactating individuals, those with significant cognitive impairments, a history of drug allergies, or those engaging in frequent vigorous physical activities, smoking, or regular alcohol consumption were also excluded. Of the 90 participants who met the inclusion criteria, none were excluded from the study. Apart from discontinuing the product containing glutathione, we make sure the eligibility and restriction criteria, detailed participant instruction, and regular monitoring thus minimize external factors that can influence research results. We have provided education that the use of skin lightening products is not permitted during the research period. These strict criteria were implemented to ensure the study accurately evaluated the efficacy and safety of SAG as a skin‐lightening agent, minimizing external influences and maintaining consistency across the participant pool. Randomization of study subjects was performed using simple randomization by a computer‐generated list with a random number generator. Subjects were grouped blindly using sealed envelopes. The envelopes consisted of 3 categories: 1 referred for SAG group, 2 referred for glutathione, and 3 referred for placebo. Double‐blinding was performed, with both participants and investigators unaware of the group assignments. We used identical packaging for the intervention products.

### 2.3 Treatment Procedure

The treatment was administered following a standardized protocol to ensure consistency across all participants. Subjects were randomly assigned to one of three groups: oral crystallin form of S‐Acetyl Glutathione (Emothion is a Registered Trademark of Gnosis S.p.A) 200 mg daily, reduced glutathione (GSH) 500 mg daily, or a placebo. S‐acetyl glutathione has been proven to have a high bioavailable rate and efficient absorption, making it more effective for oral intake. The supplementation was taken once daily for 12 weeks, this based on when administered orally, the gastrointestinal tract, primarily the jejunum, can absorb glutathione as an intact tripeptide. Significant effects are recorded within 12 weeks (3 months) of the needed amount of 20 to 40 mg/kg. To minimize external variables, participants were instructed to discontinue any use of skin‐lightening agents, antioxidants, or topical products containing glutathione at least 1 month prior to the study. All participants were provided with the same brand of mild, non‐medicated soap for personal hygiene throughout the study period.

This rigorous and systematic approach ensured a comprehensive evaluation of the efficacy and safety of SAG while controlling for external factors that could influence the outcomes.

### 2.4 Data Collection

Change of melanin index (measured by Mexameter MX18 Courage‐Khazaka, Germany) from baseline (Week 0) to end‐of‐study (Week 12) on a sun‐exposed area (face) was assessed for primary outcome. The Mexameter is a skin measurement device that quantifies melanin and erythema levels using narrow‐band light absorption, providing objective and reproducible pigmentation and redness data. Change of trans‐epidermal water loss (measured by Tewameter TM‐300; Courage‐Khazaka, Germany) from Week 0 to Week 4, 8, and 12 on a sun‐exposed area was also collected as secondary outcomes. The Tewameter is a device that measures transepidermal water loss (TEWL) to objectively assess the integrity of the skin barrier. For addition in secondary outcomes, facial skin assessment was conducted using the A‐One skin analyzer (Bomtech, Korea), a multi‐light digital imaging system for semi‐quantitative evaluation of pigmentation, with scale ranging from 0 to 10, where 0 indicates no pigmentation and 10 indicates severe pigmentation. All measurements with devices were performed in full face area including chin, cheek, and frontal area. Nine point hedonic scale was used for evaluating patient's global assessment (1 to dislike extremely, 5 to neutral and 9 to like extremely). Subjective physician's and patient's assessment with 3 category scale were also assembled. Change in total GSH in erythrocytes (measured by BIOXYTHECH GSH‐420 assay kit) from baseline to end of study was collected for exploratory outcomes. Compliance was monitored through daily treatment logs in paper form that can be reviewed regularly by study team and unused doses were verified during follow‐ups, as well as side effects. Participants were required to report any side effects, such as itching, irritation, redness, nausea, vomiting, or burning sensations, during the study. During every follow‐up session, the researchers evaluated the melanin index, TEWL index, and skin analyzer measurements, as well as monitored participants' adherence to the prescribed regimen using the logs. Standardized clinical photographs were collected at each study visit, while patient‐reported satisfaction scores were evaluated at the final follow‐up visit. Strict follow‐up adherence was maintained through regular assessments of participants' logbook documentation to minimize missing data.

### 2.5 Statistical Analysis

Data were presented as mean (±standard deviation) for continuous variables and frequency (percentage) for categorical variables. For primary outcomes and secondary outcomes that needed interaction between each group and time, we used mixed models or difference in difference (Did) analysis with the following equation = “outcome = groups + timepoint + interaction group timepoint”. With this model, we adjusted for confounding factors which are age and skin type. The relationship between age and skin type according to the Fitzpatrick Skin Phototype classification is closely associated with melanin levels, TEWL, and GSH levels as indicators of skin biological function. Individuals with higher Fitzpatrick skin types generally have greater melanin content, which provides natural protection against ultraviolet radiation. With increasing age, melanocyte activity may change, affecting the distribution and amount of melanin in the skin. Aging is also associated with a decline in epidermal barrier function due to reduced stratum corneum lipids and slower skin cell regeneration, which may lead to increased TEWL as an indicator of greater water loss through the skin. In addition, the endogenous antioxidant capacity, including GSH levels, tends to decrease with age, increasing susceptibility to oxidative stress. Therefore, age and Fitzpatrick skin type contribute to variations in melanin levels, skin barrier integrity as reflected by TEWL, and antioxidant status represented by GSH levels.

The standardized mean difference (SMD) and confidence interval were used to have better insight for analyzing treatment efficacy. Post‐test design using the Kruskal Wallis test was used for patients and physician's assessment. For exploratory outcomes, GSH levels were also analyzed with the Kruskal Wallis test. Statistical analyses were conducted using IBM SPSS Statistics 24.0 software (SPSS Inc., United States) to ensure accurate and reliable interpretation of the study results.

## 3 Results

A total of 90 subjects were assessed and enrolled. These people were randomly divided into SAG, glutathione, and placebo groups. 30 subjects in each group were included in the study. During the study, 3 subjects from the SAG group were lost to follow up (Figure 1). The basic demographics of the three groups of patients are compared. As seen, there was no significant difference between the three groups in terms of these variables (age and Fitzpatrick skin type), thus indicating homogeneity in the groups (Table 1). For significant difference in melanin index, we controlled with Difference in Difference analysis (Did).

**FIGURE 1:**
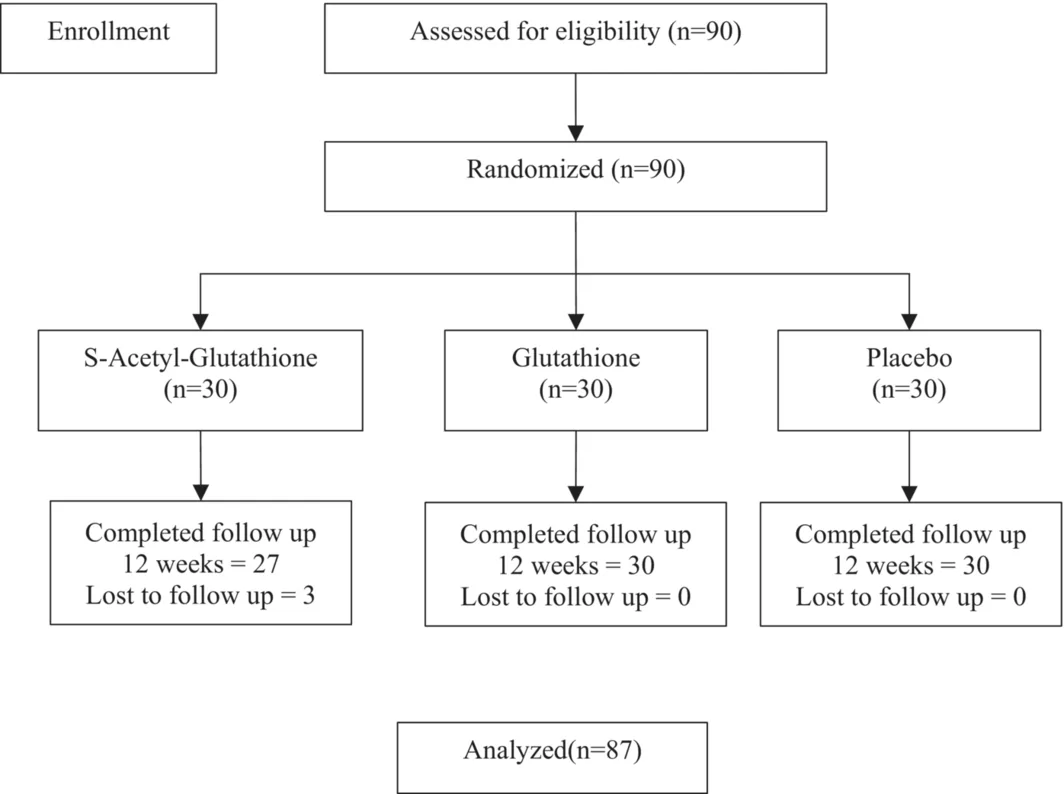
Consort flow diagram.

**TABLE 1:** Baseline characteristics of the subjects.

Variable	Placebo (*N* = 30)	Glutathione (*N* = 30)	SAG (*N* = 27)	Total (*N* = 87)	*p*
Age (years), mean ± SD	39.27 ± 9.27	39.47 ± 7.73	38.63 ± 6.59	39.14 ± 7.90	0.919
Skin type					
Fitzpatrick IV, *n* (%)	30 (100.0%)	30 (100.0%)	27 (100.0%)	87 (100.0%)	—
Fitzpatrick V, *n* (%)	0 (0.0%)	0 (0.0%)	0 (0.0%)	0 (0.0%)	—
Melanin index, mean ± SD	270.06 ± 56.49	319.53 ± 80.92	353.50 ± 107.23	313.01 ± 89.07	0.006*
TEWL, mean ± SD	20.46 ± 11.49	25.69 ± 13.49	23.63 ± 15.51	23.25 ± 13.54	0.176
GSH level, mean ± SD	666.03 ± 167.88	748.77 ± 235.74	720.78 ± 202.43	711.55 ± 204.60	0.247
Pigmentation level, mean ± SD	2.20 ± 1.51	2.13 ± 1.35	2.22 ± 1.57	2.18 ± 1.46	0.972

* *p* < 0.05.

Our primary outcomes showed no statistically significant differences in the melanin index were observed at Weeks 4, 8, and 12 within each group. However Difference‐in‐differences (DiD) analysis demonstrated that the intervention had a statistically significant effect on the melanin index based on time to time (Table 2), to enhance the analysis, another method used to calculate the effect size of SAG, those reveal compared with placebo, SAG demonstrated a large effect size in reducing the melanin index at Week 4 (SMD = 1.274, 95% CI: 0.742–1.805) and Week 8 (SMD = 0.828, 95% CI: 0.297–1.360). A similar effect size was observed with glutathione, which demonstrated a large effect size in reducing melanin levels compared with placebo at Week 4 (SMD = 0.833, 95% CI: 0.316–1.350) and Week 12 (SMD = 0.841, 95% CI: 0.324–1.358). Notably, the melanin‐lowering effect of SAG was more prominent at Week 4 than that of glutathione, suggesting an earlier onset of action of SAG in reducing melanin levels (SMD = 1.006, 95% CI: 0.474–1.537; Table S1).

**TABLE 2:** Melanin index, TEWL, pigmentation level, and GSH level comparison.

Variable	Time point	Placebo (*N* = 30)	Glutathione (*N* = 30)	SAG (*N* = 27)	Total (*N* = 87)	*p*	*p* **
Melanin index, mean ± SD							0,001**
	W0	270.06 ± 56.49	319.53 ± 80.92	353.50 ± 107.23	313.01 ± 89.07	0.006*	
	W4	297.39 ± 62.05	314.60 ± 71.40	329.13 ± 107.10	313.18 ± 81.57	0.686	
	W8	303.63 ± 62.86	301.67 ± 66.81	303.24 ± 96.01	302.83 ± 75.00	0.789	
	W12	315.93 ± 71.77	286.61 ± 63.93	289.01 ± 88.73	297.47 ± 75.35	0.253	
TEWL, mean ± SD							0.808
	W0	20.46 ± 11.49	25.69 ± 13.49	23.63 ± 15.51	23.25 ± 13.54	0.176	
	W4	19.02 ± 10.20	24.34 ± 13.36	20.22 ± 13.39	21.23 ± 12.44	0.166	
	W8	15.84 ± 6.42	22.60 ± 12.92	18.14 ± 13.23	18.88 ± 11.46	0.047*	
	W12	13.82 ± 5.58	21.35 ± 12.61	15.34 ± 11.53	16.89 ± 10.74	0.013*	
Pigmentation level, mean ± SD							0.841
	W0	2.20 ± 1.51	2.13 ± 1.35	2.22 ± 1.57	2.18 ± 1.46	0.972	
	W4	1.53 ± 1.35	1.76 ± 1.22	1.48 ± 1.39	1.59 ± 1.31	0.482	
	W8	1.36 ± 1.40	1.70 ± 1.23	1.37 ± 1.47	1.48 ± 1.36	0.335	
	W12	1.33 ± 1.37	1.70 ± 1.02	1.33 ± 1.44	1.45 ± 1.28	0.453	
GSH level, mean ± SD							
	W0	666.03 ± 167.88	748.77 ± 235.74	720.78 ± 202.43	711.55 ± 204.60	0.247	
	W12	953.93 ± 246.48	968.93 ± 276.28	897.15 ± 271.74	941.48 ± 263.60	0.477	

* *p* < 0.05.

** *p* value = DiD analysis, *p* < 0.05.

A statistically significant difference in TEWL was observed at Weeks 8 and 12 between each group, even though it did not show a significant effect on TEWL over the same time points and Did analysis (Table 2). Pigmentation values did not differ significantly among all groups, in both analyses (Table 2).

Both SAG and glutathione showed a consistent reduction in melanin index from baseline through Week 12, while all groups demonstrated comparable downward trends in TEWL and pigmentation levels over the study period, yet our exploratory outcomes showed upward trends in all groups (Figure 2a–d).

**FIGURE 2:**
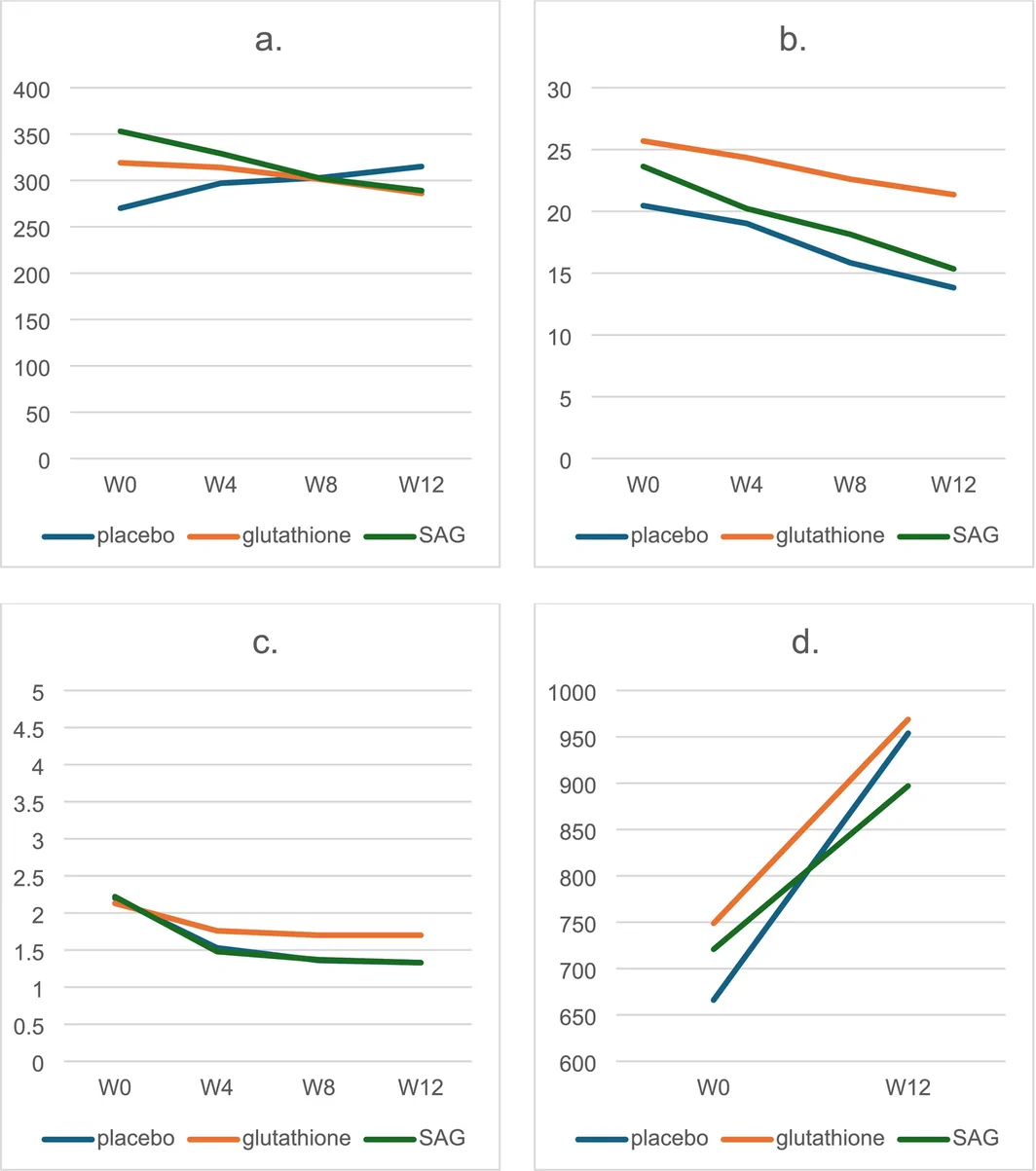
Melanin index (a), TEWL (b), pigmentation level (c), and GSH level comparison (d).

Comparative analyses confirm significant differences between SAG and both placebo and glutathione for skin lightening and skin evenness based on patients' satisfaction, SAG demonstrating the highest satisfaction followed by glutathione and placebo. Placebo showed the highest percentage in no improvement across both parameters (Table 3), aligned with doctors' satisfaction. SAG proven improving the lightening of the skin more than the placebo. These findings further establish SAG's effectiveness in enhancing skin brightness and evenness, even though we found no statistically significant difference from the hedonic scale assessment (Table 3), and exploratory outcomes reveal there are no differences in GSH levels of whole groups (Table 2).

**TABLE 3:** Physician and patients' satisfaction and hedonic scale result.

Variable	Placebo (*N* = 30)	Glutathione (*N* = 30)	SAG (*N* = 27)	*p*
Skin lightening (%)				
Very bright	3.3%	36.7%	59.3%	< 0.001*
Bright	16.7%	26.7%	29.6%	
Less bright	80.0%	36.7%	11.1%	
Skin evenness (%)				
Much improved	6.7%	33.3%	55.6%	< 0.001*
Improved	20.0%	30.0%	25.9%	
No improvement	73.3%	36.7%	18.5%	
Hedonic scale				
Like moderately	20.0%	33.3%	37.0%	0.392
Like slightly	50.0%	46.7%	51.9%	
Neither like nor dislike	30.0%	20.0%	11.1%	

* *p* < 0.05.

## 4 Discussion

This study evaluated the effects of 12 weeks of supplementation with 200 mg/day of SAG, compared to 500 mg reduced glutathione and placebo on various outcomes including melanin index, pigmentation and transepidermal water loss. The baseline demographic data show a homogeneous sample in terms of age and Fitzpatrick skin type across all groups. This uniformity minimizes variability that could potentially influence outcomes, ensuring the comparability of treatment effects. However, this homogeneity limits the generalizability of the findings to individuals with other Fitzpatrick skin types. The decision to focus on age and Fitzpatrick skin Type IV and V in this study was driven by their relevance to the treatment being evaluated. Fitzpatrick skin Type IV and V represent a population with moderate pigmentation, which is known to respond uniquely to various dermatological treatments compared to other skin types [20, 21]. By selecting this specific demographic, the study aims to explore treatment efficacy and safety in a group that often faces underrepresentation in clinical research.

The progressive increase in melanin index in the placebo group underscores the absence of active ingredients to counteract melanin production, reinforcing the benefits of active treatments like SAG. Regarding melanin index, both SAG and Glutathione resulted in pigmentation reductions, though SAG appear to be the quickest in reducing melanin index at early week. Confirmation both by objective measurement of melanin index and pigmentation showed SAG and glutathione performed better than the placebo. This help understanding the importance use of glutathione to lighten the skin in person with no skin disorders and might also be an adjuvant therapy for hyperpigmentation disorders. There still few large studies about SAG, or glutathione. Studies show that glutathione inhibits the enzyme tyrosinase, which plays a critical role in melanin production, thereby reducing hyperpigmentation. There are various administration of glutathione has been made both in topical, oral or intravenous [22]. Pigmentation disorders such as melasma can be effectively treated with glutathione due to its strong anti‐melanogenic properties [23]. Various studies have demonstrated that both oral and topical forms of glutathione result in significant improvements in skin pigmentation, as evidenced by reductions in the melanin index and overall skin tone enhancement [24]. A systematic review by Sarkar et al. [23] found that topical glutathione at a 0.5% concentration was significantly more effective in skin lightening compared to 0.1% and placebo.23 Furthermore, the combination of 2% topical glutathione with oral glutathione showed superior results compared to single treatments.

A study by Mohamed [25] showed that combining microneedling with glutathione resulted in a statistically significant greater reduction in Hemi‐mMASI scores compared to microneedling alone, with the mean score on the right side of the face (microneedling with glutathione) decreasing from 4.21 ± 2.08 to 1.96 ± 1.30, while the left side (microneedling alone) decreased from 4.06 ± 1.91 to 2.31 ± 1.45. Meanwhile, a study by Farahat et al. [26] found that both topical (2% cream) and oral glutathione (500 mg/day capsules) significantly reduced modified melasma area and severity index (MASI) scores, with *p*‐values of 0.011 and 0.007, respectively, while topical or oral placebo showed no response. These studies confirm that glutathione is a safe and effective skin lightening agent for melasma treatment.

Cystallin form of SAG has emerged as a more innovative form of glutathione, offering improved stability and bioavailability [27, 28]. Its acetylation process increases stability but also shields it from enzymatic breakdown and promotes efficient cellular absorption. Once within the cytoplasm, SAG is metabolized into active GSH, delivering powerful antioxidant effects [29]. This great bioavailability makes SAG a preferable option for managing oxidative imbalances associated with pigmentation disorders.

Reductions in TEWL were observed across all groups. While glutathione and placebo also exhibited reductions, this study highlighted SAG for its capability maintaining skin integrity. Not much large studies related to oral SAG and TEWL has been reported. Study report topical glutathione reducing TEWL at Week 10 [30], Another double blind, and RCT was performed and reported that oral reduced gluthatione lowering TEWL significantly [31]. Improvements stem from the antioxidant properties of glutathione, which reduce oxidative stress, increase collagen synthesis, and support overall skin regeneration [27]. These findings emphasize the potential of SAG not only for skin lightening, but also as a comprehensive dermatological treatment to improve skin health and appearance [28].

The observed GSH levels did not differ between groups from W0 to W12, which may be explained by several factors. First, erythrocytes function more as a storage site for GSH rather than a direct indicator of its variability. As a result, measuring GSH in erythrocytes alone may not fully capture fluctuations in its levels. Future studies could improve precision by assessing GSH levels in both plasma and erythrocytes to provide a more comprehensive analysis. Additionally, dietary intake was not strictly controlled. The consumption of GSH‐ or cysteine‐rich foods (such as meat, fish, and eggs) may have contributed to variations in GSH levels across individuals. Therefore, the individual dietary variable could have masked potential differences between groups.

A significant result of patients and physician assessment in SAG and glutathione groups indicate that the ability of SAG or glutathione to provide skin lightening and skin evenness upshot. Research by Perricone [32] also identified SAG as an effective derivative in topical formulations, with the ability to reduce melanin production, thereby providing skin lightening effects [28]. These findings position SAG derivatives as a promising alternative for patients unresponsive to traditional therapies, offering a novel approach to managing hyperpigmentation [28]. Similarly, the antioxidant potential of glutathione was highlighted in an in vitro study that evaluated the effects of a combination of SAG, *Silybum marianum* , and Vitamin E in mitigating oxidative stress on hepatic cells [29]. The findings align with SAG efficacy in skin applications, underscoring the broader potential of glutathione‐based formulations in addressing oxidative stress and enhancing cellular health across various biological systems.

### 4.1 Limitations

This study has several limitations that need to be addressed. Most existing literature focuses on conventional glutathione formulations, leaving data on the stability, bioavailability, and mechanisms of action of SAG relatively scarce. Additionally, the study population was relatively homogenous, consisting of participants with Fitzpatrick skin types IV, limiting the generalizability of the results to other demographic groups. The study's relatively short duration of 12 weeks also restricted the ability to assess the long‐term safety and efficacy of SAG. The study did not explore interactions between SAG and other commonly used skin therapy ingredients, such as combinations with additional antioxidants or skin‐lightening agents.

### 4.2 Further Research

Further research should include more diverse populations, encompassing various skin types, ages, ethnicities, and skin conditions. Long‐term studies are essential to evaluate the safety and sustained efficacy of SAG over extended periods. Additionally, deeper exploration of the molecular mechanisms of SAG, particularly its role in oxidative stress modulation, tyrosinase inhibition, and other relevant biochemical pathways, could provide more detailed insights into its mode of action.

Further studies should compare SAG with other systemic skin‐lightening agents, such as tranexamic acid, arbutin, or niacinamide, to establish its position as a preferred therapeutic option. Research on combinations of SAG with other ingredients, like vitamin C or E, could reveal potential synergies in enhancing therapeutic outcomes. Moreover, optimizing SAG formulations, including aspects like comfort, texture, and administration methods, could improve patient satisfaction and support broader adoption.

Large‐scale, multicenter clinical trials with randomized, controlled designs are needed to validate the findings of this study and strengthen the evidence base for SAG. Further research can be done in other glutathione‐based therapies, offering better solutions for managing pigmentation disorders and improving overall skin health.

## 5 Conclusion

While the findings of this study demonstrate the efficacy of SAG in skin lightening and overall skin health improvement, further research is necessary to fully establish its therapeutic potential. The experimental study of SAG compared to placebo or reduced glutathione should be further investigated, especially in the long term (> 12 weeks). These results underscore the dual role of S‐acetyl glutathione not only as a potent skin‐lightening agent but also as a supportive treatment for enhancing overall skin health. Studies with larger, more diverse populations are crucial to validate these results and explore the effects of S‐acetyl glutathione across different demographic groups, including varying skin types and conditions. Additionally, long‐term studies are essential to assess its sustained efficacy and safety over extended periods of use. Investigating its mechanism of action in greater detail, particularly its role in oxidative stress modulation and cellular health, could provide valuable insights. Comparative studies with other systemic skin‐lightening agents and topical formulations in glutathione‐based therapies might be done to find better treatment options for managing hyperpigmentation and enhancing skin health.

## Author Contributions

A.K., L.N. contribute to concept and design of the work; A.M., E.R. contribute to data acquisition; A.K., L.N., S.V., A.M., E.R. contribute to data analysis/interpretation; A.K., A.M., E.R. contribute to drafting the manuscript; L.N., S.V., A.K., A.M., E.R. contribute to critical revision of the manuscript; A.K., A.M., E.R. contribute to statistical analysis; A.K., L.N. contribute to supervision; All authors approved the final manuscript.

## Funding

This work was supported by PT Signa Husada.

## Conflicts of Interest

The authors declare no conflicts of interest.

## Supporting information


**Table S1:** Within‐group analyses of melanin index change from W0 to W4, W8, W12 (SMDs).

## Data Availability

The data that support the findings of this study are available on request from the corresponding author. The data are not publicly available due to privacy or ethical restrictions.
