# Supplementary material for: Skin Lightening and Evenness Effects of Oral Supplementation With S‐Acetyl Glutathione in Comparison With Reduced Glutathione: A Double‐Blind, Placebo‐Controlled Clinical Trial
Source: J Cosmet Dermatol. 2026 Oct 14;25(10):e71223. doi: 10.1111/jocd.71223 (PMC13669515; doi:10.1111/jocd.71223)
Supplement: Supplementary file 1 [file JOCD-25-e71223-s001.docx]

**Supplementary Table S1. Within-group analyses of melanin index change from W0 to W4, W8, W12 (SMDs)**

| **Group** | **SMDs** | **95%CI** | |
| --- | --- | --- | --- |
|  |  | **Lower** | **Upper** |
| **Placebo VS SAG** |  |  |  |
| W4-W0 | 1.274 | 0.742 | 1.805 |
| W8-W4 | 0.828 | 0.297 | 1.360 |
| W12-W8 | 0.734 | 0.202 | 1.266 |
| **Placebo VS Glutathione** |  |  |  |
| W4-W0 | 0.833 | 0.316 | 1.350 |
| W8-W4 | 0.566 | 0.049 | 1.083 |
| W12-W8 | 0.841 | 0.324 | 1.358 |
| **Glutathione VS SAG** |  |  |  |
| W4-W0 | 1.006 | 0.474 | 1.537 |
| W8-W4 | 0.621 | 0.090 | 1.153 |
| W12-W8 | -0.046 | -0.577 | 0.486 |

SMDs Test; <0.40 (low effect); 0,4≤ SMDs <0,8 (moderate effect); SMDs ≥0.8 (large effect)
